# Chronic High-Salt Diet Activates Tumor-Initiating Stem Cells Leading to Breast Cancer Proliferation

**DOI:** 10.3390/cells13110912

**Published:** 2024-05-25

**Authors:** Lisa Tucker, Umer Ali, Roy Zent, Deborah A. Lannigan, Jeffrey C. Rathmell, Venkataswarup Tiriveedhi

**Affiliations:** 1 Department of Biological Sciences, Tennessee State University, Nashville, TN 37209, USA; 2 Division of Nephrology and Hypertension, Department of Medicine, Vanderbilt University Medical Center, Nashville, TN 37232, USA; 3 Department Biomedical Engineering, Vanderbilt University, Nashville, TN 37240, USA; 4 Vanderbilt Center for Immunobiology, Department of Pathology, Microbiology, and Immunology, Vanderbilt University Medical Center, Nashville, TN 37232, USA; 5 Division of Pharmacology, Vanderbilt University, Nashville, TN 37232, USA

**Keywords:** breast cancer, tumor-initiating stem cells, cytokines, cancer biology, immunotherapy

## Abstract

Several chronic inflammatory diseases have been linked to high-salt (HS) diets. Chronic inflammation is an established causative hallmark of cancer. However, a direct role of HS diets in tumorigenesis is yet to be defined. Previous orthotopic murine breast tumor studies have shown that short-term HS diets caused inhibition of tumor growth through the activation of cytotoxic adaptive immune responses. However, there have been experimental challenges in developing a viable chronic HS-diet-based murine tumor model. To address this, we have developed a novel chronic HS diet tumor model through the sequential passaging of tumor cells in mice under HS dietary conditions. Two orthotopic murine triple-negative breast cancer models, 4T1 tumor cells injected into BALB/c mice and Py230 tumor cells injected into C57Bl/6 mice, were utilized in our study. For the HS diet cohort, prior to orthotopic injection with tumor cells, the mice were kept on a 4% NaCl diet for 2 weeks. For the regular salt (RS) diet cohort, the mice were kept on a 1% NaCl diet. Following syngeneic cancer cell injection, tumors were allowed to grow for 28 days, following which they were collected to isolate immune cell-depleted cancer cells (passage 1, P1). The tumor cells from P1 were reinjected into the next set of non-tumor-bearing mice. This procedure was repeated for three cycles (P2–P4). In P1, compared to the RS diet cohort, we observed reduced tumor kinetics in both murine tumor models on the HS diet. In contrast, by P4, there was significantly higher tumor progression in the HS diet cohort over the RS diet cohort. Flow cytometry analysis demonstrated an 8-fold increase in tumor-initiating stem cells (TISCs) from P1 to P4 of the HS diet cohort, while there were no significant change in TISC frequency with sequential passaging in the RS diet cohort. Molecular studies showed enhanced expression of TGFβR2 and CD80 on TISCs isolated from the P4 HS diet cohort. In vitro studies demonstrated that TGFβ stimulation of these TISCs increased the cellular expression of CD80 molecules. Further, the chronic HS diet selectively induced the glycolytic metabolic phenotype over the mitochondrial oxidative phosphorylation phenotype in TISCs, which is needed for the production of metabolites during tumor cell differentiation and proliferation. The infiltrating CD8 and CD4 T-lymphocytes in P4 tumors demonstrated increased expression of the immune checkpoint inhibitor (ICI) CTLA4, a known binding partner of CD80, to cause immune exhaustion and pro-tumorigenic effects. Interestingly, anti-TGFβ monoclonal antibodies (mAbs) played a synergistic role in further enhancing the anti-tumor effect of anti-CTLA4 mAb. In summary, our findings demonstrated that chronic HS diet increased the frequency of TISCs in tumors leading to blunting of cytotoxic adaptive immune responses causing tumor proliferation. Furthermore, a combination of anti-TGFβ with current ICI-based immunotherapies could exert more favorable anti-cancer clinical outcomes.

## 1. Introduction

Cancer-related mortality accounts for approximately 18.5% of all unnatural causes of death in the United States [1]. The chronic inflammatory tumor microenvironment is a well-accepted hallmark of carcinogenesis along with its deleterious impact on various therapeutic outcomes [2]. Persistent smoldering inflammation causes an enhanced release of reactive oxygen and nitrogen species, resulting in DNA damage and pro-oncogenic transcription factor dysregulation, leading to tumorigenesis, proliferation, and metastasis [3]. High salt (sodium chloride, NaCl) is a well-known mediator of chronic inflammation [4]. Previous studies in our laboratory have demonstrated that murine breast tumors, compared to the surrounding soft tissue, accumulate 30–70% higher sodium content [5]. Similarly, sodium MRI studies in breast cancer patients have demonstrated an increased tumor sodium content of up to 63% above the surrounding soft tissue [6,7,8]. However, the role of high sodium content in breast tumors is yet to be defined.

There is a divergent body of evidence suggesting the differential role of high sodium in the tumor microenvironment. Studies from our laboratory have demonstrated that human breast cancer cells when cultured under in vitro high-salt (Δ50 mM NaCl) treatment conditions enhanced cellular proliferation through initiating G0/G1 cell cycle arrest and upregulating mitosis [9,10]. This was in stark opposition to the evidence from preclinical murine xenograft models. High-salt (HS) diets suppressed tumor progression kinetics through modulation of immunosuppressive myeloid-derived suppressor cells (MDSCs) [11,12]. Similarly, we have also demonstrated that HS treatment upregulated Th1 and Th17 effector CD4+T cell immune responses leading to tumor immune elimination and decreasing tumor progression [13]. Interestingly, studies by Chen et al., utilizing a spontaneous MMTV-PyVT breast tumor model, have shown that HS diets induce tumor progression along with enhanced tumor vascularization and lung metastasis [14]. These apparently conflicting observations could be due to differences in the tumor models (xenograft vs. spontaneous) and the lack of consensus on the definition of an HS diet. It is possible that short-term HS diets activate effector immune responses, leading to tumor immune elimination of xenografts, while in spontaneous models, tumorigenesis is a result of cancer cells outplaying the early immune-elimination phase and entering a delayed immune-exhaustion phase response, overcoming the initial HS-diet-mediated effector immune-exhaustion response. To address these challenges, we developed a novel chronic HS diet murine tumor xenograft model. In this communication, we report breast tumor kinetics utilizing this novel preclinical murine model.

Tumor-initiating stem cells (TISCs) have been shown to play a critical role in resistance to chemotherapy and cancer recurrence [15]. TISCs have the capacity to stay quiescent and, at the same time, upon appropriate stimulation, could replicate and differentiate into cancer cells [16]. Studies by Al-Hajj et al. provided some of the first proof-of-concept evidence for the existence of a unique subset of CD44+CD24-TISCs in immunocompromised preclinical murine tumor models with human breast cancer cell xenografts [17]. The presence of these TISCs was later proven in multiple solid organ tumors [18,19,20,21]. Further, TISCs have been shown to exert immune privilege, due to which, during the elimination phase of tumor immune editing, they are protected from cell death, allowing them to survive until the immune-escape phase, during which they will replicate and differentiate into cancer cells along with recruiting immunosuppressive cells such as MDSCs and FoxP3+ regulatory CD4+T cells (Tregs) [22,23,24]. Studies by Miao et al. have shown that TISCs respond to transforming growth factor β (TGFβ), leading to selective upregulation of CD80 expression [25]. It has been shown that the CD80 surface molecule preferentially engages with the inhibitory protein cytotoxic T-lymphocyte associated protein 4 (CTLA4) on T-lymphocytes, leading to immune exhaustion and tumor growth [26,27]. Using our novel murine chronic HS diet model, we determined the role of TGFβ-mediated TISC activation towards breast cancer proliferation.

## 2. Materials and Methods

### 2.1. Animals and Cell Lines

Female C57Bl/6J and BALB/cJ mice were obtained from the Jackson Laboratories (Maine, ME, USA). Mice were housed in pathogen-free facilities at Vanderbilt University Medical Center. All mice were provided ad libitum food and water until they were included in our experiments. All protocols were approved by the Vanderbilt Institutional Animal Studies Committee prior to performing any murine experiments (Protocol#M1800014-00-S1900455). Mice were placed in two salt-modified-diet experimental cohorts: (i) mice on the regular salt (RS) diet were placed on 1% NaCl food (cat#TD.90229, Envigo, Madison, WI, USA); (ii) mice on the high-salt (HS) diet were placed on 4% NaCl food (cat#TD.92034 with 1% NaCl-supplemented water). The mice were kept on the HS adjusted diet two weeks prior to the injection of tumor cells, at which stage, the mice were pair-fed in both cohorts. The ten-week-old mice were injected with tumor cells.

The murine breast cancer cells, Py230 and 4T1, syngeneic for C57Bl/6J and BALB/cJ (respectively), were obtained from the American Type Culture Collection (Manassas, VA, USA). The cells were placed in a 5% CO_2_ incubator and cultured in complete RPMI1640 media supplemented with sodium bicarbonate and L-glutamine in T175 cell culture flasks. Before murine injection, the adherent cancer cells were trypsinized and isolated from flasks. Mycoplasma testing was performed to verify that the cell cultures were contamination-free. Each mice was injected with 10^5^ syngeneic breast cancer cells into the flank. The tumor volume was calculated using the formula TV = [(TW)^2^ × (TL)]/2 obtained from caliper measurements, where TV is tumor volume, TW is tumor width, and TL is tumor length [5].

For the isolation of tumor cells from explanted tumors, collagenase digestion was performed followed by filtration through 100 μm nylon mesh to prepare a single-cell suspension. The cells were then cultured in a T175 flask with complete RPMI1640 media (mentioned above). The floating cells in the supernatant were aspirated out after 24 h. The adherent cells rested in complete media for 4 more days. These adherent cells were trypsinized, washed, and isolated for reinjection into the mice for the next passage. Cells were screened for the expression of CD4, CD8, CD14, CD19, and CD20. Only cell cultures which did not have (less than 5% aggregate) the above marker-positive cells were injected into the mice, thus confirming that no immune cells were reinjected with subsequent passage.

For immunotherapy experiments, CTLA-4 (CD152) anti-mouse monoclonal antibodies (mAb) IgG2b, (clone 9D9, catalog#BE0164, BioXcell, Lebanon, NH, USA), and IgG1κ mAb against TGFβ (clone 1D11.16.8, catolog#BE0057) along with the matched isotype control (IgG2b isotype-clone MPC-11, catalog#BE0086; IgG1κ isotype-clone MOPC-21, catalog#BE0083) were utilized. Mice were injected with 200 µg of mAb on days 7, 10, and 13. All chemicals were obtained from Sigma-Aldrich (St. Louis, MO, USA) or Fisher Scientific (Waltham, MA, USA), unless mentioned otherwise.

### 2.2. Isolation of TISCs and CD8+ and CD4+ T Cells

The breast-tumor-bearing C57Bl/6J and BALB/cJ mice were sacrificed on day 28 post-injection of breast cancer cells to obtain tumor tissue. A single-cell preparation was prepared from these harvested tissues. The TISCs were isolated from murine tumors by CD24^−^CD44^+^ breast tumor stem cells using immunomagnetic beads as per the manufacturer’s specifications and reagents (catalog#MAGH111, R&D Systems, Minneapolis, MN, USA). The TISCs were isolated by negative selection for CD24 expression, followed by positive selection for CD44 expression. The isolated TISCs were cultured in complete RPMI1640 media until functional metabolic studies were performed on these cells. The CD8+T cells were isolated from the single-cell suspension by negative selection to isolate untouched CD8+T cells (catalog#19753, STEMcell technologies, Cambridge, MA, USA). Similarly, CD4+T cells were isolated from the single-cell suspension by negative selection to obtain untouched CD4+T cells (catalog#19752, STEMcell technologies, Cambridge, MA, USA). The purified CD8+ and CD4+T cells were suspended in complete RPMI1640 media until further functional studies were performed.

### 2.3. Flow Cytometry

Single-cell suspensions from tumor tissue were utilized for flow cytometry analysis [13]. Single-cell suspensions were blocked for non-specific antibody binding by incubating cells with 12.5 μg/mL of mouse IgG (12.5 μg/mL diluted in PBS on ice) for 30 min. The cells were stained with fluorophore-labeled antibodies. We utilized the following antibody panel for the gating strategy: CD24-PE (catalog#553262, clone#M1/69, BD Biosciences, Franklin Lakes, NJ, USA), CD44-APC (catalog#561862, clone#IM7, BD Biosciences), CD8-PE (catalog#561095, clone#53-6.7, BD Biosciences), CD4-FITC (catalog#553729, clone#GK1.5, BD Biosciences), CD80-PerCP-Cy5.5 (catalog#560526, clone#16-10A1, BD Biosciences), 7-AAD (catalog#559925, BD Biosciences), pSMAD2/3-PE (catalog#559925, BD Biosciences), and TGFβR2-PE (catalog# FAB532P, polyclonal, R&D Systems). Other flow cytometry dyes such as PKH26 and PI were obtained from Sigma-Aldrich (St Louis, MO, USA). Isotype-matched control monoclonal antibodies were used to determine the efficiency of FcR blocking. For intracellular staining, appropriate permeabilization buffers were used as per the manufacturer’s recommendation. The fluorescence minus one (FMO) controls were used to set the gating strategy. The cell suspensions were stained with propidium iodide (PI, 5 μg/mL) immediately before flow analysis. Data were acquired using the BD LSR Fortessa flow cytometer (BD Bioscience, Franklin Lakes, NJ, USA) and analyzed using FlowJo V10 software (FlowJo LLC, Ashland, OR, USA).

### 2.4. Quantitative Real-Time Polymerase Chain Reaction (RT-PCR)

Gene expression profiles were analyzed using the TaqMan FAM-labeled RT-PCR primers (Applied Biosystems/Thermo Fisher Scientific, Grand Island, NY, USA) for cadherin1 (Mm01247357_m1), Snail2 (Mm00441531_m1), Aldh1A1 (Mm00657317_m1), Sox2 (Mm03053810_s1), ITGA6 (Mm00434375_m1), granzyme B (Mm00442837_m1), interferon-γ (Mm01168134_m1), LAG3 (Mm00493071_m1), CTLA4 (Mm00486849_m1), PD1 (Mm01285676_m1), TIM3 (Mm01294183_m1), GADPH (Mm00467257_m1), and actin (Mm02619580_g1), as per the manufacturer’s recommendation [10]. RNA was extracted and reverse transcribed. The reaction was performed at a 20 μL final volume in triplicates with 40 cycles using BioRad CFX96 (Hercules, CA, USA).

### 2.5. Cytotoxicity Assay

The cytolytic efficacy of CD8+ and CD4+T cells, analyzed based on their ability to lyse the target breast cancer cells, was calorimetrically determined by a non-irradiative lactate dehydrogenase (LDH) release assay (Promega, Madison, WI, USA) and also by a flow cytometry-based cytotoxic assay. Briefly, for the LDH release assay [28], 1 × 10^4^ target cells (syngeneic murine breast cancer cells) were plated in round-bottom 96-well plates in quadruplicate along with CD8+ or CD4+T cells (effector cells) at an effector to target (E:T) ratio of 20:1. The percentage of lysis was calculated using the following formula: [(experimental LDH release − spontaneous LDH release)/(maximum LDH release − spontaneous LDH release)] × 100. The flow cytometer-based cytotoxicity assay will provide further evidence for cancer-cell-specific lysis; immune cell death is not accounted for in this calculation. Towards this, cancer cells are first stained with a cell membrane fluorophore PKH26 followed by treatment with immune cells (CD8+ or CD4+ T cells). The cancer cell target lysis is measured by PHK26+7AAD+ vents in flow cytometry analysis.

### 2.6. Metabolic Assays

The metabolic activity of TISCs isolated by magnetic immunobeads (mentioned above) was studied by analyzing the extracellular acidification rate (ECAR) and oxygen consumption rate (OCR) using the Seahorse XF HS mini (Agilent, Santa Clara, CA, USA) [29]. The acidification rate was analyzed by sequential addition of glucose (100 mM), oligomycin (2.5 μM), and 2-deoxy glucose (500 mM). Glycolytic capacity was analyzed based on increases in the ECAR following the application of oligomycin above the baseline ECAR reading on 15,000 TISCs cultured in basal DMEM with no additional glucose or glutamine. The difference in the ECAR between the oligomycin injection and glucose injection was utilized to calculate apparent glycolytic reserve capacity. Apparent respiratory reserve capacity was defined as the percentage increase in OCR between the initial baseline measurement on 15,000 TISCs cultured in basal DMEM with an additional 100 mM of glucose, followed by the injection of 500 nM of the ionophore FCCP (p-trifluoromethoxy carbonyl cyanide phenyl hydrazone), an uncoupler of oxidative phosphorylation and electron transport. Glycolysis was inhibited by 500 mM 2DG, and mitochondrial complexes I and III were inhibited by rotenone (50 nM)/antimycin A (100 nM), respectively.

### 2.7. Statistical Analysis

Data are expressed as mean ± SEM (standard error of mean). Statistical significance with *p* < 0.05 between groups was assessed using Tukey HSD pair-wise comparisons for two groups and one-way ANOVA with post hoc Bonferroni correction or one-way ANOVA with Dunnett’s test for multiple comparisons. All data analysis was carried out using Prism 8 (GraphPad software, Boston, MA, USA), SPSS software version 21 (IBM corporation, Armonk, NY, USA), or Origin 6 software (Origin Labs, Northampton, MA, USA).

## 3. Results

### 3.1. Novel Chronic High-Salt Tumor Model: Enhanced Tumor Kinetics with Serial Passaging of Cancer Cells following High-Salt-Diet Treatment

**Figure 1:**
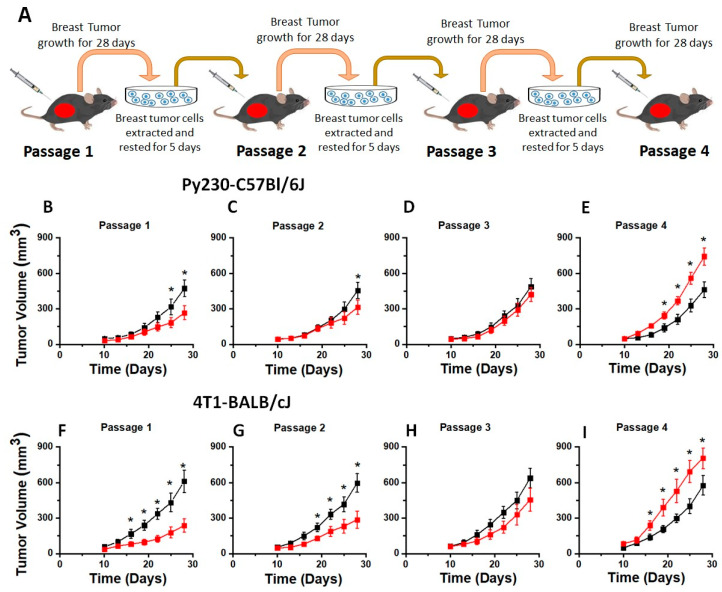
Tumor kinetics in our novel orthotopic chronic HS diet murine tumor model. (**A**) To maintain the tumor cells in a chronic HS tumor microenvironment, the tumor cells were sequentially passaged four times. In passage 1, the syngeneic breast cancer cells (Py230 or 4T1) were injected into mice breast pads (C57Bl/6J or BALB/cJ, respectively). For the HS diet cohort, the mice were kept on an HS diet 2 weeks prior to the injection of tumor cells. At the time of the diet switch, the HS diet cohort mice were pair-fed with the RS diet cohort mice. The tumors were allowed to grow for 28 days following injection, at which point, the mice were sacrificed and tumors were explanted. The harvested tumors were digested with collagenase and tumor cells were isolated. The tumor cells isolated from the RS diet cohort were rested and cultured in regular complete RPMI1640 media, while those isolated from the HS diet cohort were cultured in complete media supplemented with salt (Δ50 mM NaCl) for 5 days. The supernatant media containing floating non-tumor and dead cells were aspirated 24 h after isolation, at which time fresh medium (noted above) was added to incubate the cells at 37 °C for another 4 days. The adherent cells were then trypsinized and isolated for injection into passage 2 mice. This process was repeated until passage 4 mice. (**B**–**I**) Tumor volume changes following four murine passages in RS (■1% NaCl) and HS (■4% NaCl) dietary conditions. Tumor volume kinetics followed for 28 days in two murine breast tumor models: (**B**–**E**) C57Bl/6J mice injected with orthotopic Py230 breast tumor cells, and (**F**–**I**) BALB/cJ mice injected with orthotopic 4T1 breast tumor cells. Data presented as mean ± SEM; *n* = 8; statistical analysis was performed using two-way ANOVA multiple comparisons and Dunnet’s post-test (*) *p*-value < 0.05.

Previous HS-diet-based tumor studies performed by various research groups involved switching the mice to a salt-modified diet at the time of tumor injection to 2 weeks prior to injection [5,11,12]. However, these dietary modifications could be considered short-term (acute) changes. Diet-induced chronic inflammatory responses in human diseases are a result of chronic HS dietary intake for several years. Developing a chronic HS diet murine tumor model could be challenging. For example, murine tumors, as per IACUC approval, should be halted when tumors reach a size of 1.5 cm. Further, mice carrying a tumor burden for more than 60–80 days develop ulceration or inflammatory sickness as marked by redness of the eyes. Therefore, chronic HS-diet-based murine tumor experiments (3–6 months) were difficult to perform. To overcome this experimental limitation, and at the same time subjecting the tumor cells to chronic HS dietary treatment conditions, we developed a unique chronic HS diet model by sequentially passaging tumor cells in mice kept on an HS diet. Towards this, mice were injected in the flank with 3 × 10^5^ syngeneic murine breast tumor cells (Figure 1A). For the HS dietary cohort, mice were fed a high-salt diet for 2 weeks (14 days) prior to the injection of tumor cells. As shown in Figure 1B, when Py230 breast cancer cells were injected into C57Bl/6J mice, the HS cohort showed reduced tumor progression (day 28, 267 ± 59 mm^3^) compared to the regular salt diet (RS) cohort (day 28, 473 ± 72 mm^3^, *p* < 0.05). These data were in line with studies from other laboratories, including our previous published results [5,12]. The tumors were explanted on day 28, harvested, and digested to isolate tumor cells. The floating cells in the culture flask, predominantly consisting of immune cells and dead cells, were aspirated and removed after 24 h. The adherent tumor cells were cultured for four more days. Only the cells sticking to the culture plate, depleted of macrophages, leukocytes, and lymphocytes, were utilized for further injection into mice (Figure 1C–E). Tumors from the HS cohort were cultured in complete media supplemented with 50 mM of sodium chloride (Δ50 mM NaCl) and injected into mice kept on an HS diet for 2 weeks prior to injection. As shown in Figure 1E, by passage 4, enhanced tumor progression was noticed in the HS diet cohort (day 28, 741 ± 83 mm^3^ vs. RS: 459 ± 67 mm^3^, *p* < 0.05). Similar results were obtained in a BALB/cJ murine model injected with 4T1 syngeneic breast tumor cells under the same treatment conditions. Previous data from our laboratory and others have shown that high-salt diets in mice induced the activation of anti-tumor T-lymphocytes. These data, along with our current findings, suggest that in passage 1, the decreased tumor progression in the HS diet cohort was due to the immune elimination of tumor cells by high-salt-mediated inflammatory activation of effector T-lymphocytes. However, enhanced tumor growth by passage 4 suggested that tumor cells became immune-resistant and escaped immune elimination. Tumor-initiating stem cells (TISCs) represent a subset of tumor cells which are known to play a critical role in acquired resistance and tumorigenicity [30]. Therefore, based on the data from Figure 1, we hypothesized that following the chronic HS diet (by passage 4), there was expansion of TISCs playing a critical role and causing enhanced tumor progression.

### 3.2. Enhanced TISC Frequency in Tumors following Chronic HS Diet

**Figure 2:**
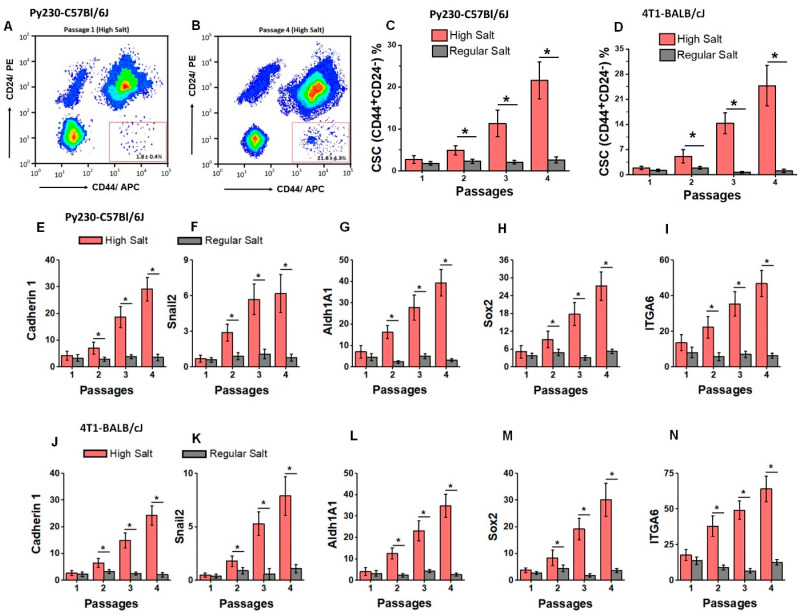
Enumeration of TISC frequency in various murine passages following dietary salt modification. The frequency of CD44+CD24−TISCs cells in the isolated tumor cells among RS (grey) and HS (red) diet cohorts was enumerated by flow cytometry. (**A**,**B**) Representative flow cytometry plot of TISCs from passage 1 and passage 4 of Py230-C57Bl/6J HS diet cohort. (**C**,**D**) Changes in TISC frequency with each passage in Py230-C57Bl/6J (**C**) and 4T1-BALB/cJ (**D**) murine tumor models. (**E**–**N**) The mRNA expression of TISC markers Cadherin 1 (**E**,**J**), Snail2 (**F**,**K**), Aldh1A1 (**G**,**L**), Sox2 (**H**,**M**), and ITGA6 (**I**,**N**) in four passages of Py230-C57Bl/6J and 4T1-BALB/cJ tumor models (respectively). Data analyzed by one-way ANOVA for multiple comparisons and presented as mean ± SEM; *n* = 8 (biological replicates) per cohort; (*) *p*-value < 0.05.

We determined the frequency of TISCs in our HS diet tumor model by flow cytometry analysis of CD44+C24- cells. Tumors from day 28 in each passage were harvested, digested, isolated, and cultured for 5 days as mentioned above. The floating cells and debris were discarded. The sticky cells were analyzed for surface expression of CD44 and the lack of a presence of the CD24 marker. As shown in Figure 2A–C, in the Py230-C57Bl/6J murine model, there was an expansion of CD44+C24- cells in the HS diet cohort by passage 4 (from 2.7 ± 0.9% in passage 1 to 21.6 ± 4.4% in passage 4, *p* < 0.05). However, there was no significant expansion of CD44+C24-cells in the RS diet cohort (1.8 ± 0.4% in passage 1 to 2.6 ± 0.7% in passage 4, *p* > 0.05). A similar expansion of CD44+C24- cells in the passage 4 HS cohort was noted in the 4T1-BALB/cJ murine model. We next assessed the expression of TISC-specific genes in the explanted tumor cells by qRT-PCR [31]. As shown in Figure 2E–I, in the Py230-C57Bl/6J model, we noted 8.6-fold enhanced expression of Cadherin1, 10.3-fold enhanced expression of Snail2, 8.4-fold enhanced expression of Aldh1A1, 7.2-fold enhanced expression of Sox2, and 5.7-fold enhanced expression of ITGA6 in the passage 4 HS cohort compared to the passage 1 HS cohort. No significant change in the expression of the above genes was observed between passage 1 and passage 4 in the RS cohort. Further, as shown in Figure 2J–N, a similar enhanced expression profile of the above-mentioned genes was also noted in the 4T1-BALB/cJ model. These data demonstrated that sequential passaging of tumor cells in our novel chronic HS diet tumor model caused an expansion of TISCs, accounting for its higher tumorigenicity.

### 3.3. Cytotoxic Efficiency of Tumor-Infiltrating CD8+T Cells

**Figure 3:**
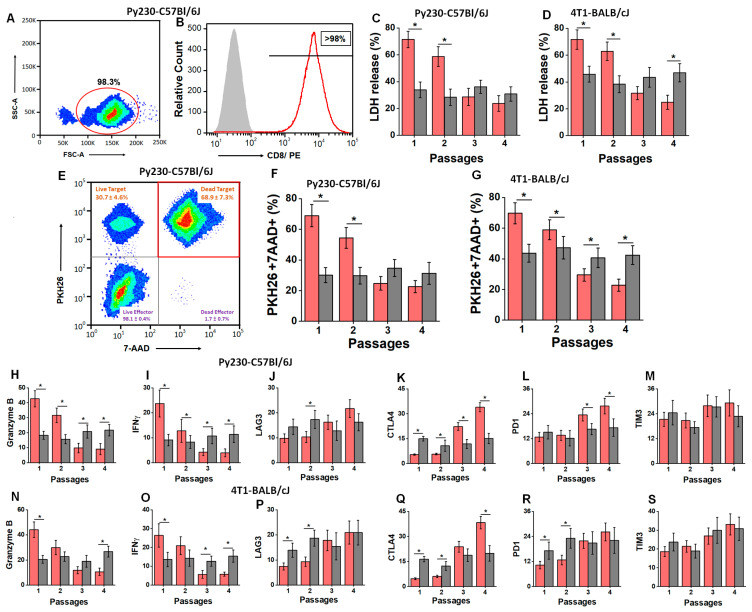
Anti-tumor efficiency of tumor-infiltrating CD8+T cells. (**A**,**B**) CD8+T cells were isolated by negative selection using magnetic immunobeads. The FSC/SSC plot shows that the isolation yielded >95% purity, which was further confirmed by staining with CD8-PE; isotype control shown in grey and CD8+T cells shown in red. (**C**,**D**) Cytotoxic efficiency of CD8+T cells on the passage-matched syngeneic cancer cells (E:T ratio 20:1) isolated from the explanted tumors from RS (grey) and HS (red) diet cohorts was assessed by LDH release assay in Py230-C57Bl/6J (**C**) and 4T1-BALB/cJ (**D**) murine tumor models. (**E**) Representative flow cytometry-based analysis of cytotoxicity with labeling of PKH26 labeled cancer cells. The dead cell events were measured by 7-AAD/PKH26 double-positive events. (**F**,**G**) Cytotoxic efficiency of CD8+T cells on the passage-matched syngeneic cancer cells (E:T ratio 20:1) isolated from the explanted tumors from RS (grey) and HS (red) diet cohorts as assessed by PKH26+7-AAD+ events in Py230-C57Bl/6J (**F**) and 4T1-BALB/cJ (**G**) murine tumor models. (**H**–**S**) The mRNA expression of inflammatory cytokines: granzyme B (**H**,**N**) and IFNγ (**I**,**O**); exhaustion/inhibitory markers: LAG3 (**J**,**P**), CTLA4 (**K**,**Q**), PD1 (**L**,**R**), and TIM3 (**M**,**S**) in the tumor-infiltrating CD8+T cells from each of the four passages in RS (grey) and HS (red) diet cohort of Py230-C57Bl/6J and 4T1-BALB/cJ tumor models (respectively). Data analyzed by one-way ANOVA for multiple comparisons and presented as mean ± SEM; *n* = 8 (biological replicates) per cohort; (*) *p*-value < 0.05.

As enhanced frequency of TISCs in passage 4 is associated with increased tumor progression, we next analyzed if there were changes in the cytotoxic efficiency of tumor-infiltrating CD8+T cells. As shown in Figure 3A,B, tumor-infiltrating CD8+T cells were isolated by negative selection using magnetic beads, and the purity was confirmed to be >95%. The cytotoxic efficiency of tumor-infiltrating CD8+T cells was determined on passage-matched tumors. In the Py230-C57Bl/6J model (Figure 3C), passage 4 tumor-infiltrating CD8+T cells (at E:T ratio 20:1) from the HS cohort exerted significantly reduced cytotoxicity (23.8 ± 5.7%) compared to their passage 1 counterparts (71.4 ± 6.1%; *p* < 0.05). A similar reduction in anti-tumor cytotoxicity (passage 1, 70.6 ± 7.2% to passage 4, 24.9 ± 5.2%; *p* < 0.05) was observed in the HS diet cohort mice from the 4T1-BALB/cJ model. However, the anti-tumor cytotoxicity of tumor-infiltrating CD8+T cells did not change among all four passages in the RS diet cohort from both murine tumor models. To further confirm the specific cytotoxicity against target cells, we labeled the target cells (tumor cells) with PKH26. The double-positive events (PKH26 and 7-AAD) were determined to be dying tumor cells due to the cytotoxic effect of effector cells (CD8+T cells). As shown in Figure 3F,G, 98% of the 7AAD+ events came from PKH26 labeled cells, indicating that the observed cytotoxicity was from tumor cell death and not from effector CD8+T cells. We further verified the expression of various inflammatory and exhaustion markers in tumor-infiltrating CD8+T cells by qRT-PCR. As shown in Figure 3H–M, in the HS diet cohort of the Py230-C57Bl/6J murine model, we observed a 4.7-fold decrease in granzyme B, a 6-fold decrease in IFNγ, a 6.3-fold increase in CTLA4, and a 2.1-fold increase in PD1 from passage 1 to passage 4, while the expression of LAG3 and TIM3 did not show a statistically significant difference between passage-matched HS and RS cohorts. Similarly, in the HS diet cohort of the 4T1-BALB/cJ murine model, we observed significantly decreased expression of inflammatory genes (granzyme B and IFNγ) along with increased expression of immune-exhaustion marker CTLA4 from passage 1 to passage 4. However, we did not observe a statistically significant difference in the expression of PD1, TIM3, and LAG3 between passage-matched HS and RS cohorts from this murine model. These data clearly demonstrated that sequential passaging of tumors under HS dietary conditions is associated with decreased inflammatory response and increased immune exhaustion, and thus the blunting of CD8+T cell anti-tumor responses.

### 3.4. Anti-Tumor Efficiency of Tumor-Infiltrating CD4+T Cells

**Figure 4:**
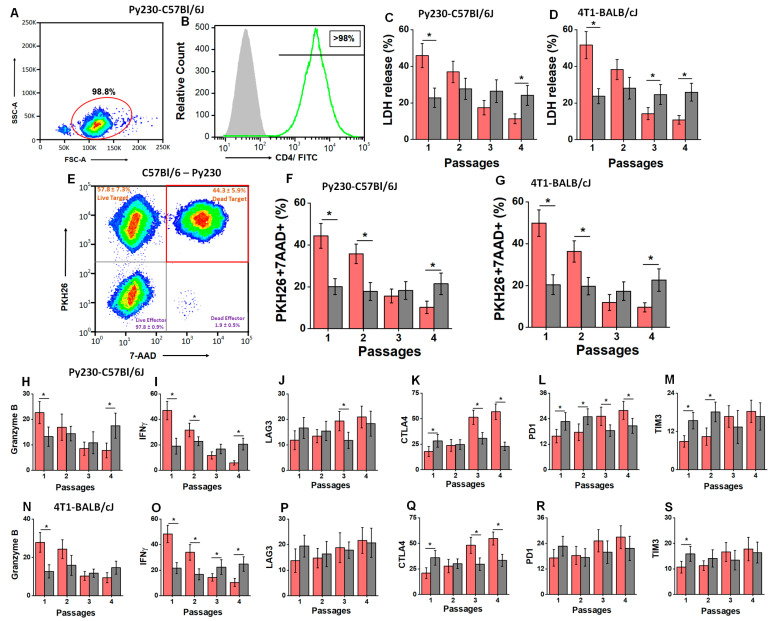
Anti-tumor efficiency of tumor-infiltrating CD4+T cells. (**A**,**B**) CD4+T cells were isolated by negative selection using magnetic immunobeads. The FSC/SSC plot shows that the isolation yielded >95% purity, which was further confirmed by staining with CD4-FTIC; isotype control shown in grey and CD4+T cells shown in green. (**C**,**D**) Cytotoxic efficiency of CD4+T cells on the passage-matched syngeneic cancer cells (E:T ratio 20:1) isolated from the explanted tumors from RS (grey) and HS (red) diet cohorts as assessed by LDH release assay in Py230-C57Bl/6J (**C**) and 4T1-BALB/cJ (**D**) murine tumor models. (**E**) Representative flow cytometry-based analysis of cytotoxicity with labeling of PKH26 labeled cancer cells. The dead cell events were measured by 7-AAD/PKH26 double-positive events. (**F**,**G**) Cytotoxic efficiency of CD4+T cells on the passage-matched syngeneic cancer cells (E:T ratio 20:1) isolated from the explanted tumors from RS (grey) and HS (red) diet cohorts as assessed by PKH26+7-AAD+ events in Py230-C57Bl/6J (F) and 4T1-BALB/cJ (**G**) murine tumor models. (**H**–**S**) The mRNA expression of inflammatory cytokines: granzyme B (**H**,**N**) and IFNγ (**I**,**O**); exhaustion/inhibitory markers: LAG3 (**J**,**P**), CTLA4 (**K**,**Q**), PD1 (**L**,**R**), and TIM3 (**M**,**S**) in the tumor-infiltrating CD4+T cells from each of the four passages in RS (grey) and HS (red) diet cohorts of Py230-C57Bl/6J and 4T1-BALB/cJ tumor models (respectively). Data analyzed by one-way ANOVA for multiple comparisons and presented as mean ± SEM; *n* = 8 (biological replicates) per cohort; (*) *p*-value < 0.05.

As we noticed decreased anti-tumor efficiency of CD8+T cells from passage 1 to 4, we next tested for the anti-tumor efficiency of CD4+T cells in our chronic HS diet model. The tumor-infiltrating CD4+T cells were isolated (Figure 4A,B) by magnetic beads, and the purity was confirmed to be >95%. The anti-tumor efficiency of tumor-infiltrating CD4+T cells was determined by an LDH release assay and flow cytometry on passage-matched tumor cells. As shown in Figure 4C, the tumor-infiltrating CD4+T cells isolated from the HS diet cohort of the Py230-C57Bl/6J murine model demonstrated that there was reduced cytotoxicity against tumor cells (E:T ratio 20:1) from passage 1 (45.7 ± 6.6%) to passage 4 (11.3 ± 2.7%; *p* < 0.05). Similarly, there was diminished anti-tumor cytotoxicity (passage 1, 51.6 ± 7.4% to passage 4, 10.8 ± 2.3%; *p* < 0.05) in the HS diet cohort of the 4T1-BALB/cJ murine model. However, the anti-tumor efficiency of CD4+T cells did not change among all four passages in the RS cohort of both murine models. As mentioned above, we further confirmed the specific cytotoxicity of target cells over effector cells by labeling the target cells with PKH26. As shown in Figure 4F,G, 98% of the 7AAD+ events came from PKH26 labeled cells, confirming that the observed cytotoxicity was from tumor cell death and not from effector CD4+T cells. Examination of the expression of inflammatory and exhaustion markers in tumor-infiltrating CD4+T cells by qRT-PCR (Figure 4H–M) demonstrated a 2.9-fold decrease in granzyme B, an 8.1-fold decrease in IFNγ, a 3.2-fold increase in CTLA4, an 1.8-fold increase in PD1, a 2.1-fold increase in TIM3, and a 1.7-fold increase in LAG3 from passage 1 to passage 4 in the HS diet cohort of the C57Bl/6J murine model. Similarly, in the HS cohort of the BALB/cJ murine model, we observed significantly decreased expression of inflammatory genes (granzyme B and IFNγ) along with increased expression of immune-exhaustion marker CTLA4 from passage 1 to passage 4. These data suggest that, similar to CD8+T cell responses, sequential passaging of tumors under HS dietary conditions is associated with decreased inflammatory response and increased immune exhaustion of tumor-infiltrating CD4+T cells.

### 3.5. TGFβ-Mediated Expression of CD80 on TISCs

**Figure 5:**
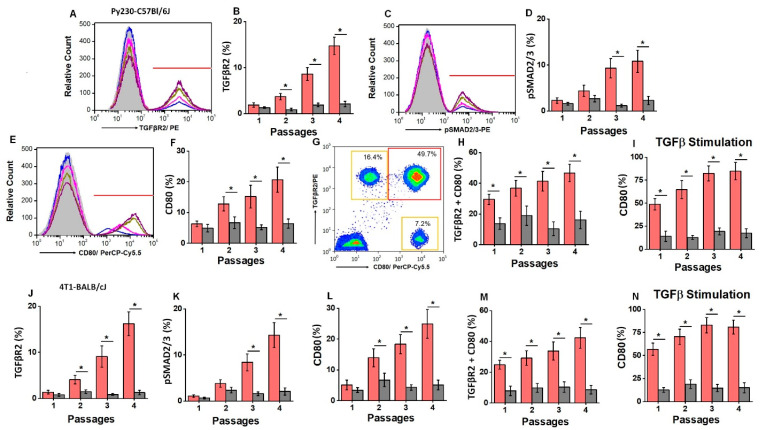
TGFβ signaling induced the expression of immune-inhibitory CD80 on TISCs. (**A**) Representative flow cytometry histogram of TGFβR2 expression in passages 1 (blue), 2 (pink), 3 (olive green), and 4 (purple) from the HS diet cohort of the Py230-C57Bl/6J tumor model. (**B**) Comparison of the relative expression of TGFβR2 in each of the four passages on CD4+CD24-TISCs isolated from RS (grey) and HS (red) diet cohorts of the Py230-C57Bl/6J tumor model. (**C**) Representative flow cytometry histogram of pSMAD2/3 intracellular expression in each of the four passages from the HS diet cohort of the Py230-C57Bl/6J tumor model. (**D**) Comparison of the relative expression of pSMAD2/3 in each of the four passages on CD4+CD24-TISCs isolated from the RS and HS diet cohorts of the Py230-C57Bl/6J tumor model. (**E**) Representative flow cytometry histogram of CD80 surface expression in each of the four passages from the HS diet cohort of the Py230-C57Bl/6J tumor model. (**F**) Comparison of the relative expression of CD80 in each of the four passages on CD4+CD24-TISCs isolated from the RS and HS diet cohorts of the Py230-C57Bl/6J tumor model. (**G**) Representative flow cytometry plot to determine the TGFβR2/CD80 double-positive TISCs in passage 4 of the HS diet cohort from the Py230-C57Bl/6J tumor model. (**H**) Comparison of the relative expression of TGFβR2/CD80 double-positive TISCs in each of the four passages on CD4+CD24-TISCs isolated from the RS and HS diet cohorts of the Py230-C57Bl/6J tumor model. (**I**) Comparison of the relative expression of CD80 following TGFβ (80 ng/mL) stimulation for 5 days on CD4+CD24-TISCs isolated from each of the four passages of the RS and HS diet cohorts in the Py230-C57Bl/6J tumor model. (**J**–**M**) Comparison of the relative expression of TGFβR2 (**J**), pSMAd2/3 (**K**), CD80 (**L**), and TGFβR2/CD80 double-positive cells (**M**) in each of the four passages on CD4+CD24-TISCs isolated from the RS (grey) and HS (red) diet cohorts of the 4T1-BALB/cJ tumor model. (**N**) Comparison of the relative expression of CD80 following TGFβ (80 ng/mL) treatment for 5 days on CD4+CD24-TISCs isolated from each of the four passages of the RS and HS diet cohorts of the 4T1-BALB/cJ tumor model. Data analyzed by one-way ANOVA for multiple comparisons and presented as mean ± SEM; *n* = 8 (biological replicates) per cohort; (*) *p*-value < 0.05.

Studies from other laboratories have shown that TISCs have higher expression of transforming growth factor (TGF)-β receptor (TGFβR2). Further, TGFβ upregulates the expression of CD80, which enables TISCs to engage with CTLA4 on CD4+T cells, leading to the inhibition of effector immune responses. To verify this, we analyzed the expression of TGFβR2 in the TISCs obtained from our newly developed murine tumor model. As shown Figure 5A,B, the CD44+CD24-TISCs isolated from the Py230-C57Bl/6J murine model demonstrated increased expression of TGFβR2 from passage 1 (1.9 ± 0.6%) to passage 4 (14.7 ± 1.8%; *p* < 0.05) in the HS diet cohort. Further, TGFβ signaling molecule pSMAD2/3 expression (Figure 5C,D) was also upregulated from passage 1 (2.3 ± 0.7%) to passage 4 (10.8 ± 2.4%; *p* < 0.05) in the HS diet cohort. As CD80 expression was shown by others to be induced by TGFβ, we next analyzed the expression of CD80. As shown in Figure 5E,F, CD80 expression was also upregulated from passage 1 (6.3 ± 0.9%) to passage 4 (20.7 ± 4.1%; *p* < 0.05) in the HS diet cohort. Further, we observed that 29.5 ± 3.4% of TISCs in the HS diet cohort co-expressed both TGFβR2 and CD80 in passage 1 (Figure 5G,H), which increased to 46.7 ± 5.8% (*p* < 0.05) by passage 4. However, in the RS diet cohort, there was no change in the expression levels of TGFβR2 and CD80 during sequential passages. To test for the direct role of TGFβ in CD80 expression, we stimulated TISCs with TGFβ in vitro. As shown in Figure 5I, following the TGFβ stimulation of TISCs isolated from the HS diet cohort, there was increased expression of CD80 in passage 1 (48.9 ± 6.3%, post-treatment vs. 6.3 ± 0.9%, pre-treatment, *p* < 0.05). Similarly, there was increased CD80 expression following TGFβ treatment in passage 4 (84.7 ± 9.4%, post-treatment vs. 20.7 ± 4.1%, pre-treatment, *p* < 0.05). Similar results were also obtained in the 4T1-BALB/cJ breast tumor model (Figure 5J–N). Taken together, these data clearly demonstrated that the chronic HS diet resulted in TGFβ-mediated cell surface expression of CD80 in TISCs, which is associated with immune exhaustion and tumor progression.

### 3.6. Co-Treatment with Anti-CTLA4 and Anti-TGFβ Monoclonal Antibodies in HS Cohort Reduced Tumor Progression

**Figure 6:**
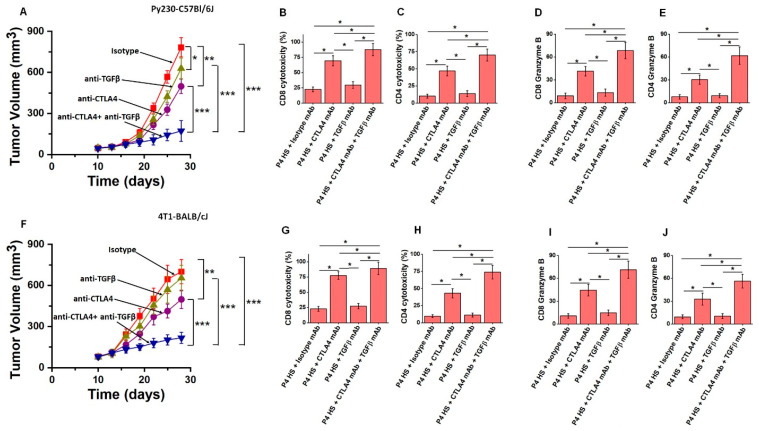
Combinatorial treatment of anti-TGFβ with anti-CTLA4 mAb resulted in enhanced anti-tumor impact. (**A**,**F**) Tumor volume changes in passage 4 of the chronic HS diet cohort kept on treatment with isotype mAb (red), anti-TGFβ mAb (olive green), anti-CTLA4 mAb (purple), and anti-CTLA4 mAb+anti-TGFβ mAb (blue) in the Py230-C57Bl/6J (**A**) and 4T1-BALB/cJ (**F**) tumor models. The tumor growth was analyzed with a mixed-effects model on the natural log scale to better meet normality assumptions. Data presented as mean ± SE of tumor volume over time; n = 8 per treatment group. The *p*-values for multiple comparisons were adjusted using the Holm–Bonferroni method; (*) *p* < 0.05, (**) *p* < 0.01, and (***) *p* < 0.001. (**B**,**G**) Cytotoxicity of tumor-infiltrating CD8+T cells isolated on day-28 tumors from the four treatment cohorts (mentioned above) against the tumor cells (at E:T ratio 20:1) isolated from passage 4 of the HS diet cohort without treatment as assessed by LDH release assay in Py230-C57Bl/6J (**B**) and 4T1-BALB/cJ (**G**) tumor models. (**C**,**H**) Cytotoxicity of tumor-infiltrating CD4+T cells isolated on day 28 from the four treatment cohorts against the tumor cells (at E:T ratio 20:1) isolated from passage 4 of the HS diet cohort without treatment as assessed by LDH release assay in Py230-C57Bl/6J (**C**) and 4T1-BALB/cJ (**H**) tumor models. (**D**,**I**) The mRNA expression of granzyme B in CD8+T cells isolated on day 28 from the four treatment cohorts in passage 4 of the HS diet cohort of Py230-C57Bl/6J (**D**) and 4T1-BALB/cJ (**I**) tumor models. (**E**,**J**) The mRNA expression of granzyme B in CD4+T cells isolated on day 28 from the four treatment cohorts in passage 4 of the HS diet cohort of Py230-C57Bl/6J (**E**) and 4T1-BALB/cJ (**J**) tumor models. Data analyzed by one-way ANOVA for multiple comparisons and presented as mean ± SEM; *n* = 8 (biological replicates) per cohort; (*) *p*-value < 0.05.

Studies from other laboratories have shown that CD80 expression on TISCs in the context of CTLA4 on immune cells results in a blunted effector anti-tumor response and promotes tumor progression [32]. As our studies have shown TGFβ-mediated upregulation of CD80 expression following the sequential passaging of tumor cells in the HS cohort, we next studied if the combination of anti-TGFβ monoclonal antibodies (mAbs) with immune the checkpoint inhibitor anti-CTLA4 mAb will impact tumor progression. As shown in Figure 6A, in passage 4 of the HS diet cohort from the Py230-C57Bl/6J model, the combination of anti-TGFβ mAb with anti-CTLA4 mAb significantly reduced the tumor progression (day 28, 172 ± 76 mm^3^ vs. 783 ± 104 mm^3^, isotype control mAb, *p* < 0.05) compared to individual anti-CTLA4 mAb treatment (day 28, 498 ± 53 mm^3^, *p* < 0.05) or anti-TGFβ mAb treatment alone (day 28, 629 ± 76 mm^3^, *p* < 0.05). Further, analysis of the anti-tumor cytotoxicity (E:T ratio 20:1) of CD8+ and CD4+T cells demonstrated a 3.9- and 6.8-fold increased response (respectively, Figure 6B,C) following combinatorial treatment of anti-CTLA4 mAb with anti-TGFβ mAb, compared to isotype treatment. Further, this combinatorial treatment increased cytotoxic cytokine (granzyme B) expression. We also noted a similar response in the 4T1-BALB/cJ murine breast tumor model following the combination of anti-TGFβ mAb with anti-CTLA4 mAb (Figure 6F–J). Taken together, these data suggest that chronic HS treatment upregulated TGFβ-mediated immune exhaustion, leading to tumor progression, and the combination of immune checkpoint inhibitor therapy (anti-CTLA4 mAb) with anti-TGFβ mAb is more efficient at reducing tumor progression.

### 3.7. Chronic High Salt Induced the Glycolytic Metabolic Pathway

**Figure 7:**
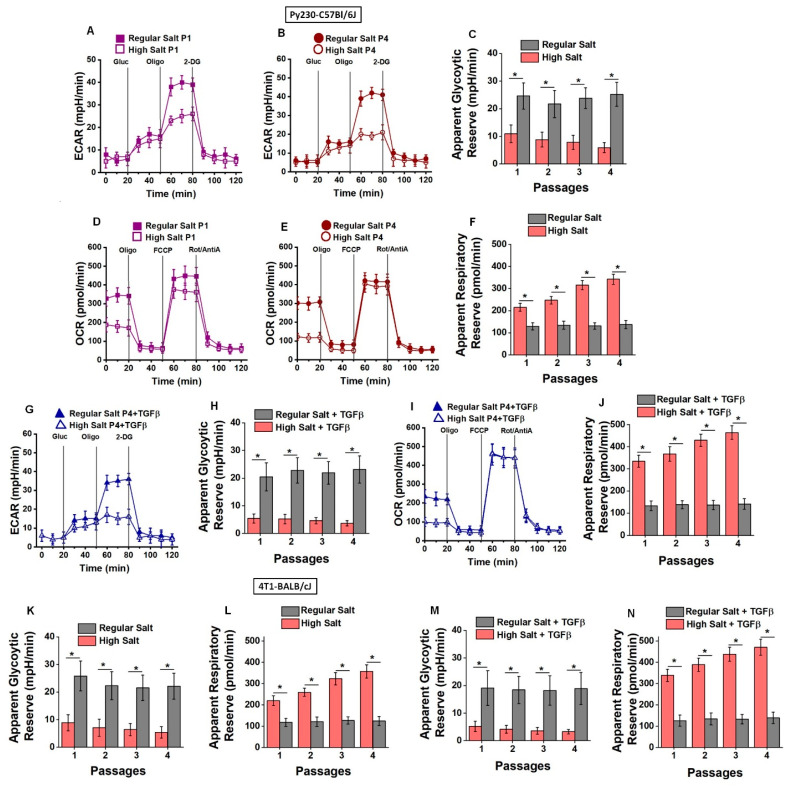
Chronic HS diet induced a metabolic phenotype switch in TISCs. The TISCs obtained from explanted tumors in each of the four passages in RS and HS dietary cohorts were cultured in base DMEM media without glucose and glutamine. pH-sensitive measurement of extracellular acidification rate (ECAR, **A**–**C**) was assessed by time-course changes in lactate production following addition of 100 mM glucose (gluc), mitochondrial inhibitor oligomycin (oligo, 2.5 μM), and glycolysis inhibitor 2-deoxy glucose (2DG, 500 mM). (**A**) Passage 1 TISCs from RS cohort (Py230-C57Bl/6J) demonstrated increased glycolytic activity following treatment with mitochondrial inhibitor (oligo) compared to HS diet counterparts, suggesting that TISCs from HS diet cohort were already at saturation capacity of glycolysis which could not be further enhanced by oligo treatment. (**B**) Passage 4 TISCs from RS cohort also demonstrated increased glycolytic activity following treatment with mitochondrial inhibitor (oligo) compared to HS diet counterparts, suggesting that TISCs from HS diet cohort were already at saturation capacity of glycolysis which could not be further enhanced by oligo treatment. (**C**) Comparisons in the glycolytic reserve capacity among TISCs from each of the four passages demonstrated that RS diet cohort had higher glycolytic reserve compared to HS diet cohort, suggesting that glycolysis is the major metabolic pathway in TISCs under HS dietary conditions. For measuring oxygen consumption rate (OCR, **D**–**F**) to determine the mitochondrial metabolism, the TISCs were cultured in base DMEM media with 100 mM glucose for basal OCR response, followed by time-course response changes with addition of mitochondrial inhibitors (oligo, 2.5 μM), proton gradient disruptor (carbonyl cyanide-4-(trifluoromethoxy) phenylhydrazone, FCCP, 500 nM) and rotenone/antimycin A (Rot/AntiA, 50/100 nM). (**D**) Passage 1 TISCs from RS cohort (Py230-C57Bl/6J) demonstrated increased basal OCR over HS cohort counterparts, suggesting that TISCs from RS cohort have oxidative phosphorylation (OXPHOS) metabolic phenotype. Following treatment with metabolic inhibitors (oligo, FCCP, Rot/AntiA), TISCs from both cohorts demonstrated a similar change in OCR pattern. (**E**) Passage 4 TISCs from RS cohort (Py230-C57Bl/6J) demonstrated increased basal OCR over HS cohort counterpart, suggesting that TISCs from RS cohort have oxidative phosphorylation (OXPHOS) metabolic phenotype. (**F**) Comparison of apparent respiratory reserve capacity among both cohorts demonstrated that TISCs from HS diet cohort in all four passages have higher OXPHOS reserve over their RS counterparts, suggesting that while OXPHOS is the major metabolic phenotype in RS cohort, glycolysis is the major metabolic phenotype in HS cohort. (**G**,**H**) ECAR studies following stimulation with TGFβ demonstrated that HS diet cohort maintained glycolytic phenotype. Although we noticed a slight decrease in glycolytic reserve capacity in HS+TGFβ cohort (**H**) versus HS alone (**C**), this difference did not reach statistical significance. Similarly, we observed slight decreases in glycolytic reserve between RS+TGFβ (**H**) vs. RS alone (**C**), which was also was not statistically significant. These data suggested that TGFβ further slightly enhanced the glycolytic metabolic phenotype (although statistically insignificant) in TISCs from both RS and HS cohorts. (**I**,**J**) OCR studies following stimulation with TGFβ demonstrated that RS diet cohort maintained OXPHOS phenotype. The respiratory reserve capacity of all 4 passages of HS+TGFβ (**J**) versus HS alone (**F**) showed statistically significant differences, suggesting that TGFβ further reduced the OXPHOS metabolism in chronic HS diet, leading to enhanced glycolytic metabolism, which is needed to produce additional macromolecules associated with cancerous cell differentiation and proliferation in Py230-C57Bl/6J tumor model. (**K**–**N**) The glycolytic reserve changes (**K**) and respiratory reserve changes (**L**); alongside these changes, after TGFβ treatment (**M**,**N**), the 4T1-BALB/cJ model followed a similar pattern to the Py230-C57Bl/6J model mentioned above. Data analyzed by one-way ANOVA for multiple comparisons and presented as mean ± SEM; *n* = 6 (biological replicates) per cohort; (*) *p*-value < 0.05.

All our above-mentioned data strongly suggested that the chronic HS diet induced TISCs, leading to tumorigenesis. As tumor cells are known to preferentially upregulate the glycolytic pathway over the oxidative phosphorylation pathway to produce the metabolic intermediates needed for growth and proliferation by a phenomenon known as the Warburg effect [33,34], we next tested if the upregulation of TISCs in our chronic high-salt model was also associated with a similar metabolic shift in these cells. The CD44+CD24-TISCs isolated from explanted tumors were utilized for extracellular metabolic analysis using the Seahorse XF HS Mini (Agilent, Santa Clara, CA, USA). The glycolytic stress analysis was performed by measuring lactate production by TISCs, which was determined by changes in the extracellular acidification rate (ECAR) following sequential treatment with 100 mM of glucose, oligomycin, and 2-deoxy-D-glucose (2-DG). As shown in Figure 7A,B, TISCs from the RS diet cohort of passage 1 Py230-C57Bl/6J tumor-bearing mice demonstrated that following treatment with oligomycin, which inhibits mitochondrial ATP production and shifts the TISCs to glycolytic metabolism, there was an enhanced ECAR (39 ± 3.7 mpH/min) compared to the passage-matched HS diet cohort (24.6 ± 3.2 mpH/min, *p* < 0.05). Similarly, we observed an enhanced ECAR in passage 4 in the RS diet cohort (41.2 ± 3.4 mpH/min) compared to the HS diet cohort 20.1 ± 2.7 mpH/min (*p* < 0.05), following treatment with oligomycin. Further, there was a 4.3-fold reduced apparent glycolytic reserve in TISCs by passage 4 in the HS diet cohort (Figure 7C) compared to the passage-matched RS diet cohort, suggesting that the TISCs from the HS cohort were utilizing the glycolytic pathway at their highest saturating capacity and thus had a decreased glycolytic reserve capacity. Studies to analyze oxidative metabolism demonstrated that (Figure 7D–F) the baseline oxygen consumption rate (OCR) in passage 1 in the RS diet cohort was higher (338 ± 41 pmol/min) than the HS diet cohort (179 ± 38 pmol/min, *p* < 0.05). Similarly, OCR in passage 4 of the RS diet cohort was higher (303 ± 32 pmol/min) than the HS diet cohort (119 ± 22 pmol/min, *p* < 0.05). Further, the apparent respiratory reserve capacity in passage 4 of the HS diet cohort was 2.6-fold higher than in the RS diet cohort. This OCR data clearly demonstrated that TISCs from the HS diet cohort did not utilize the oxidative phosphorylation (OXPHOS) pathway at its full capacity.

To determine the effect of TGFβ stimulation on metabolic shift, TISCs were treated with TGFβ in vitro for 3 days followed by measurement of glycolytic stress and OCR. As shown in Figure 7G,H, TGFβ stimulation induced a reduction in the apparent glycolytic reserve capacity in passage 4 of the HS diet cohort (3.7 ± 0.9 mpH/min) compared to the RS diet cohort (41.2 ± 3.4 mpH/min, *p* < 0.05). Conversely, the apparent respiratory reserve capacity in the TGFβ-stimulated passage 4 HS diet cohort demonstrated a 3.3-fold increase over the RS diet cohort, suggesting that TGFβ stimulation further enhanced the pro-Warburg-like metabolic shift in TISCs. Similar findings were observed in the 4T1-BALB/cJ model (Figure 7K–N). Taken together, the OCR and glycolytic stress data clearly demonstrated that the chronic HS diet induced TISCs to upregulate the anabolic pathways needed for eventual tumor cell differentiation and proliferation.

## 4. Discussion

HS diets are associated with several cardiovascular and inflammatory diseases resulting in ischemic injury and end-organ stress [35,36,37]. An average Western diet is considered to have a 2–3 times higher salt content than the American Heart Association daily recommendation [38]. Studies by Wu et al. have shown that HS diets induce Th17 responses, leading to the upregulation of inflammatory autoimmune disease-causing pathways [39]. ^23^Na-MRI studies, in both murine breast tumor models and human breast cancer patients, have found a 30–70% higher tumor sodium content above the surrounding soft tissue [6,40,41]. Previous studies from our laboratory have demonstrated that HS diets cause a further 40% increase in tumor sodium concentration [5]. While cell culture studies have shown breast cancer cell proliferation following high-salt treatment [10], murine models have shown reduced breast tumor progression in HS diet cohorts [12]. Towards this, in our current studies, we have developed a novel chronic HS diet murine tumor model with sequential passaging of breast tumors for four cycles in HS diet mice. As shown in Figure 1, our chronic HS diet model demonstrated enhanced tumor progression by passage 4. These data clearly suggest a varied role of high tumor sodium content in cancer immune editing [42]. Based on this, we propose that, during the initial phase, high sodium accumulation in tumors induces effector activation of immune responses, leading to the immune-mediated elimination of tumor cells; however, in the later phases, tumor cells acquire resistance, enabling escape from immune responses and finally resulting in tumor progression. Unlike the previous short-term HS diet studies in orthotopic models, our chronic HS diet model showed tumor progression by passage 4. It is interesting to note that short-term HS diet in spontaneous breast tumor model demonstrated tumor progression. In the spontaneous MMTV-PyVT breast tumor model [14], it is possible that even a short-term HS diet will cause tumor immune elimination; however, by the time the tumor is formed in this model, the tumor has already passed through that phase of immune editing and reached the immune-escape phase, resulting in tumor growth and proliferation. Further, it is difficult to establish chronic HS diet conditions in a single passage, as prolonged tumor load in mice causes ulceration and pain, which prohibits the ethical continuation of experiments. Future studies with other non-metastatic cell lines such as 4T07, 168FARN, or 67NR will substantiate our current tumor kinetics data to analyze the localized growth of tumors under HS dietary conditions.

TISCs have been shown to play a critical role in treatment resistance and recurrence. The otherwise quiescent TISCs, following appropriate external stimulus or epigenetic changes, acquire a tumor-initiating capability leading to cancerous differentiation and full-blown cancer [43]. Our current data have shown that a chronic HS diet induced up to a 10-fold increased frequency of TISCs, along with acquiring enhanced cell surface expression of TGFβ-receptor and CD80 molecules. Based on previous evidence [44], we analyzed the expression of various TISC markers such as Cadherin1, Snail2, Aldh1A1, Sox2, and ITGA6. The expression of all these markers was enhanced by passage 4 of our chronic HS model. Studies from other laboratories have shown that TGFβ induces the expansion and differentiation of TISCs [25]. CD80 is shown to engage with the T cell inhibitory molecule CTLA4, leading to immune suppression and the promotion of tumor growth [26]. Our current data strongly support the notion that chronic HS diets cause the activation of TISCs, leading to tumorigenesis along with CD80-mediated escape of anti-tumor immune responses. Furthermore, the lack of changes in tumor kinetics with subsequent passages in the RS diet cohort could be explained by the apparent lack of changes in TISC frequency from passages 1 to 4.

**Figure 8:**
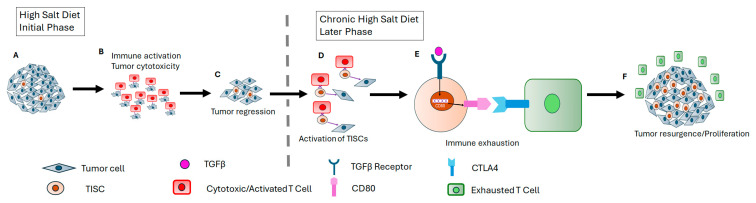
Schematic representation of the mechanistic role of chronic HS diet in tumorigenesis. (**A**) Intratumor accumulation of high salt during the early phase of tumorigenesis. (**B**) High salt activates cytotoxic immune responses. (**C**) Early-phase immune-elimination response causing tumor regression. (**D**) During chronic high-salt conditions, there is an enhanced frequency of TISCs in the tumor. These TISCs, due to their immune privilege, can evade cytotoxic immune responses and differentiate into tumor cells. (**E**) The TISCs under the influence of TGFβ cytokines induce cell surface expression of CD80 molecule. The CD80 on TISCs selectively binds with CTLA4 on T-lymphocytes causing immune exhaustion and blunting of cytotoxic responses. (**F**) In this delayed phase of high-salt conditions, a cumulative impact of increased frequency of TISCs and immune exhaustion results in resurgence, causing tumorigenesis.

Previous short-term HS diet studies have shown reduced tumor progression along with enhanced effector immune responses. Studies by Willibrand et al. and He et al. have demonstrated that short-term HS diets induce the inhibition of immune-suppressive MDSCs, leading to enhanced anti-tumor responses and resulting in reduced tumor progression [11,12]. Similarly, previous studies from our laboratory have also shown that a short-term HS diet induced interferon γ (IFNγ)/Th1-mediated anti-tumor effector immune responses [5,13]. However, our current chronic HS diet treatment has shown a blunting of effector anti-tumor immune responses, as evidenced by the reduced expression of cytotoxic granzyme B and IFNγ in CD8+ and CD4+T cells (Figure 3 and Figure 4). Our immune cell and TISC studies could provide an important perspective on our tumor kinetics data. Analysis of the tumor kinetics in the C57Bl/6-Py230 HS cohort demonstrated that, compared with the tumor volume on day 28 in passage 1 of the HS cohort, there was a 20% increase in passage 2, a 92% increase in passage 3, and a 238% increase in passage 4. Tumor growth is a combination of three independent factors, namely the anti-tumor effect of immune cells, the pro-tumor effect of TISCs, and the blunting of the cytotoxicity of immune cells. For example, compared with passage 1, there was a 1.8-fold increase in TISC in passage 2, a 4.2-fold increase in passage 3, and an 8.1-fold increase in passage 4, while the cytotoxic efficiency of CD4+T cells decreased by 18% in passage 2, by 60% in passage 3, and by 67% in passage 4; similarly, the cytotoxic efficiency of CD8+T cells decreased by 21% in passage 2, by 64% in passage 3, and by 68% in passage 4. The combination of all these factors could have resulted in enhanced tumor growth in passage 4. Along with these, there could be other factors like MDSCs, macrophage phenotype switching, etc., which require future studies. These chronic HS dietary conditions were also associated with enhanced expression of inhibitory CTLA4 on tumor-infiltrating T-lymphocytes. Future immunostaining studies to assess the immune cell activity of these tumor-infiltrating lymphocytes could further consolidate our current findings. Enhanced CD80 expression on TISCs might engage with inhibitory CTLA4 on T cells, resulting in immunosuppression and tumor progression. Our studies have shown that chronic HS diets upregulated the expression of CTLA4 in tumor-infiltrating CD8+ and CD4+T cells in both murine models. However, other exhaustion molecules such as PD1, TIM3, and LAG3 experienced significant changes in their expression profiles between the two murine models that we tested in this study. A schematic representation of the mechanistic role of chronic HS diets on tumorigenesis is provided in Figure 8.

CTLA4-based immune checkpoint inhibitors have significantly changed the landscape of breast cancer therapy [45]. However, clinical studies have shown only 18–40% therapeutic success with anti-CTLA mAb therapy [46]. Further treatment with anti-CTLA mAb has shown a higher incidence of systemic inflammatory side-effects including cardiotoxicity [47]. CTLA-4 expressed on T cells competes between the costimulatory molecule CD28 and the inhibitory molecule CD80. CTLA4 has higher affinity and avidity for CD80 over CD28. Upon binding with CD80, the effector cytotoxic response of T cells is blunted. Studies by Miao et al. have demonstrated that TGFβ in the tumor microenvironment upregulates the expression of CD80 in TISCs [25]. These data are in line with our current observations that the expression of CD80 is upregulated in TISCs, which is mediated by TGFβ stimulation. Co-treatment of anti-TGFβ mAbs with anti-CTLA4 mAbs significantly reduced the tumor progression compared to treatment with anti-CTLA4 alone (Figure 6). This could offer a novel therapeutic combination to enhance the clinical success in cancer patients.

Cancer cells are thought to upregulate the glycolysis metabolic pathway for ATP production even in an oxygen environment to increase the production of the metabolites that are needed for cancer cell growth and proliferation by a process called the Warburg effect [48]. Previous studies in our laboratory have demonstrated that HS culture conditions on human breast cancer cell lines would further augment this Warburg phenomenon [34]. TISCs have been shown to display the oxidative phosphorylation (OXPHOS) metabolic phenotype, rendering them quiescent in the tumor [49]. However, to date, there are minimal studies showing metabolic phenotype changes in TISCs following appropriate stimuli to expand and differentiate into cancer cells. Our current studies have demonstrated that chronic HS diets will switch TISCs from OXPHOS metabolism to the glycolytic phenotype (Figure 7). This could possibly explain why the initial quiescent TISCs adopt OXPHOS metabolism for ATP generation; however, following chronic HS-mediated tumor progression, TISCs acquire the Warburg metabolic phenotype, resulting in the production of the intermediates needed for differentiation and proliferation into cancer cells.

Our current studies are primarily focused on determining the role of TISCs in chronic HS dietary conditions. In humans, HS diets would be consumed over several years before the manifestation of chronic inflammatory consequences. However, as mentioned above, the murine tumor model utilized in our study has methodological limitations to directly studying the temporal impact of chronic HS diets in single murine passages. Our future work will be focused on determining the detailed changes in the immune footprint from passages 1 to 4 in our chronic HS model. Further, although our current work is focused on breast tumors, we envision similar outcomes in other solid organ tumors which require further study. Our chronic HS diet model specifically focuses on the tumor cell side of the tumor microenvironment. Immune cells undergo different changes in the chronic HS tumor microenvironment. Our current model is limited to studying tumor-infiltrating immune cells from the host, and does not test for sequential passaging of immune cells. Future investigations are required to study the tumor impact of immune cells under chronic HS diet conditions. Previous clinical human studies, including preclinical murine studies in our laboratory, have shown that anti-CTLA4 mAb treatment is associated with heightened systemic inflammatory side-effects including cardiotoxicity and death. In future, it is important to ascertain the systemic inflammatory side-effect profile and impact on metastasis from our proposed therapeutic combination of anti-TGFβ mAbs with anti-CTLA4 mAbs. In addition, further studies are warranted to check the impact of CD80 blockade on TISC-targeted anti-cancer therapy.

## 5. Conclusions

In conclusion, based on our newly developed chronic HS diet model, along with previous studies, we propose that high salt in the tumor microenvironment initially causes effector immune responses, causing anti-tumor immune elimination; however, in the later stages, HS induces otherwise quiescent TISCs to proliferate, resulting in enhanced tumor progression and immune escape. Along with cancer, chronic inflammation is associated with other diseases such as hypertension. Our novel chronic HS diet model could add future perspectives to study the crosslink between hypertension and cancer. In our murine breast tumor models, the combination of anti-TGFβ with anti-CTLA4 significantly reduced tumor progression over treatment with anti-CTLA4 alone. This could form a basis for future clinical studies to determine the potential beneficial impact of this combination on breast cancer patients. Further, as TISCs are shown to play a critical role in chronic HS-diet-mediated tumor progression, the development of specific TISC-targeted drugs could help improve breast cancer therapeutic approaches.

## Data Availability

The original contributions presented in the study are included in the article, further inquiries can be directed to the corresponding author.

## References

[1] Siegel R.L., Giaquinto A.N., Jemal A. (2024). Cancer statistics, 2024. CA Cancer J. Clin..

[2] Hanahan D. (2022). Hallmarks of Cancer: New Dimensions. Cancer Discov..

[3] Hibino S., Kawazoe T., Kasahara H., Itoh S., Ishimoto T., Sakata-Yanagimoto M., Taniguchi K. (2021). Inflammation-Induced Tumorigenesis and Metastasis. Int. J. Mol. Sci..

[4] Basdeki E.D., Kollias A., Mitrou P., Tsirimiagkou C., Georgakis M.K., Chatzigeorgiou A., Argyris A., Karatzi K., Manios Y., Sfikakis P.P. (2021). Does Sodium Intake Induce Systemic Inflammatory Response? A Systematic Review and Meta-Analysis of Randomized Studies in Humans. Nutrients.

[5] Khandekar D., Dahunsi D.O., Manzanera Esteve I.V., Reid S., Rathmell J.C., Titze J., Tiriveedhi V. (2022). Low-Salt Diet Reduces Anti-CTLA4 Mediated Systemic Immune-Related Adverse Events while Retaining Therapeutic Efficacy against Breast Cancer. Biology.

[6] Zaric O., Farr A., Minarikova L., Lachner S., Asseryanis E., Nagel A.M., Weber M., Singer C.F., Trattnig S. (2021). Tissue Sodium Concentration Quantification at 7.0-T MRI as an Early Marker for Chemotherapy Response in Breast Cancer: A Feasibility Study. Radiology.

[7] James A.D., Leslie T.K., Kaggie J.D., Wiggins L., Patten L., Murphy O’Duinn J., Langer S., Labarthe M.C., Riemer F., Baxter G. (2022). Sodium accumulation in breast cancer predicts malignancy and treatment response. Br. J. Cancer.

[8] Ouwerkerk R., Jacobs M.A., Macura K.J., Wolff A.C., Stearns V., Mezban S.D., Khouri N.F., Bluemke D.A., Bottomley P.A. (2007). Elevated tissue sodium concentration in malignant breast lesions detected with non-invasive 23Na MRI. Breast Cancer Res. Treat..

[9] Amara S., Ivy M.T., Myles E.L., Tiriveedhi V. (2016). Sodium channel gammaENaC mediates IL-17 synergized high salt induced inflammatory stress in breast cancer cells. Cell Immunol..

[10] Amara S., Majors C., Roy B., Hill S., Rose K.L., Myles E.L., Tiriveedhi V. (2017). Critical role of SIK3 in mediating high salt and IL-17 synergy leading to breast cancer cell proliferation. PLoS ONE.

[11] He W., Xu J., Mu R., Li Q., Lv D.L., Huang Z., Zhang J., Wang C., Dong L. (2020). High-salt diet inhibits tumour growth in mice via regulating myeloid-derived suppressor cell differentiation. Nat. Commun..

[12] Willebrand R., Hamad I., Van Zeebroeck L., Kiss M., Bruderek K., Geuzens A., Swinnen D., Corte-Real B.F., Marko L., Lebegge E. (2019). High Salt Inhibits Tumor Growth by Enhancing Anti-tumor Immunity. Front. Immunol..

[13] Tiriveedhi V., Ivy M.T., Myles E.L., Zent R., Rathmell J.C., Titze J. (2021). Ex Vivo High Salt Activated Tumor-Primed CD4+T Lymphocytes Exert a Potent Anti-Cancer Response. Cancers.

[14] Chen J., Liu X., Huang H., Zhang F., Lu Y., Hu H. (2020). High salt diet may promote progression of breast tumor through eliciting immune response. Int. Immunopharmacol..

[15] Khandekar D., Amara S., Tiriveedhi V. (2019). Immunogenicity of Tumor Initiating Stem Cells: Potential Applications in Novel Anticancer Therapy. Front. Oncol..

[16] Yang L., Shi P., Zhao G., Xu J., Peng W., Zhang J., Zhang G., Wang X., Dong Z., Chen F. (2020). Targeting cancer stem cell pathways for cancer therapy. Signal Transduct. Target. Ther..

[17] Al-Hajj M., Wicha M.S., Benito-Hernandez A., Morrison S.J., Clarke M.F. (2003). Prospective identification of tumorigenic breast cancer cells. Proc. Natl. Acad. Sci. USA.

[18] Zhang L., Chen W., Liu S., Chen C. (2023). Targeting Breast Cancer Stem Cells. Int. J. Biol. Sci..

[19] Romeo H.E., Barreiro Arcos M.L. (2023). Clinical relevance of stem cells in lung cancer. World J. Stem Cells.

[20] Lathia J.D., Mack S.C., Mulkearns-Hubert E.E., Valentim C.L., Rich J.N. (2015). Cancer stem cells in glioblastoma. Genes. Dev..

[21] Koukourakis I.M., Platoni K., Kouloulias V., Arelaki S., Zygogianni A. (2023). Prostate Cancer Stem Cells: Biology and Treatment Implications. Int. J. Mol. Sci..

[22] Li L., Jensen R.A. (2023). Understanding and Overcoming Immunosuppression Shaped by Cancer Stem Cells. Cancer Res..

[23] Galassi C., Musella M., Manduca N., Maccafeo E., Sistigu A. (2021). The Immune Privilege of Cancer Stem Cells: A Key to Understanding Tumor Immune Escape and Therapy Failure. Cells.

[24] Lei M.M.L., Lee T.K.W. (2021). Cancer Stem Cells: Emerging Key Players in Immune Evasion of Cancers. Front. Cell Dev. Biol..

[25] Miao Y., Yang H., Levorse J., Yuan S., Polak L., Sribour M., Singh B., Rosenblum M.D., Fuchs E. (2019). Adaptive Immune Resistance Emerges from Tumor-Initiating Stem Cells. Cell.

[26] Vackova J., Polakova I., Johari S.D., Smahel M. (2021). CD80 Expression on Tumor Cells Alters Tumor Microenvironment and Efficacy of Cancer Immunotherapy by CTLA-4 Blockade. Cancers.

[27] Zhang B., Dang J., Ba D., Wang C., Han J., Zheng F. (2018). Potential function of CTLA-4 in the tumourigenic capacity of melanoma stem cells. Oncol. Lett..

[28] Babaer D., Zheng M., Ivy M.T., Zent R., Tiriveedhi V. (2019). Methylselenol producing selenocompounds enhance the efficiency of mammaglobin-A peptide vaccination against breast cancer cells. Oncol. Lett..

[29] Gerriets V.A., Kishton R.J., Nichols A.G., Macintyre A.N., Inoue M., Ilkayeva O., Winter P.S., Liu X., Priyadharshini B., Slawinska M.E. (2015). Metabolic programming and PDHK1 control CD4+ T cell subsets and inflammation. J. Clin. Investig..

[30] Zhou H., Tan L., Liu B., Guan X.Y. (2023). Cancer stem cells: Recent insights and therapies. Biochem. Pharmacol..

[31] Walcher L., Kistenmacher A.K., Suo H., Kitte R., Dluczek S., Strauss A., Blaudszun A.R., Yevsa T., Fricke S., Kossatz-Boehlert U. (2020). Cancer Stem Cells-Origins and Biomarkers: Perspectives for Targeted Personalized Therapies. Front. Immunol..

[32] Chikuma S. (2017). CTLA-4, an Essential Immune-Checkpoint for T-Cell Activation. Curr. Top. Microbiol. Immunol..

[33] Thompson C.B., Vousden K.H., Johnson R.S., Koppenol W.H., Sies H., Lu Z., Finley L.W.S., Frezza C., Kim J., Hu Z. (2023). A century of the Warburg effect. Nat. Metab..

[34] Amara S., Zheng M., Tiriveedhi V. (2016). Oleanolic Acid Inhibits High Salt-Induced Exaggeration of Warburg-like Metabolism in Breast Cancer Cells. Cell Biochem. Biophys..

[35] Jaques D.A., Wuerzner G., Ponte B. (2021). Sodium Intake as a Cardiovascular Risk Factor: A Narrative Review. Nutrients.

[36] Hengel F.E., Benitah J.P., Wenzel U.O. (2022). Mosaic theory revised: Inflammation and salt play central roles in arterial hypertension. Cell Mol. Immunol..

[37] Schmidt-Pogoda A., Strecker J.K., Liebmann M., Massoth C., Beuker C., Hansen U., Konig S., Albrecht S., Bock S., Breuer J. (2018). Dietary salt promotes ischemic brain injury and is associated with parenchymal migrasome formation. PLoS ONE.

[38] Mente A., O’Donnell M., Rangarajan S., Dagenais G., Lear S., McQueen M., Diaz R., Avezum A., Lopez-Jaramillo P., Lanas F. (2016). Associations of urinary sodium excretion with cardiovascular events in individuals with and without hypertension: A pooled analysis of data from four studies. Lancet.

[39] Wu C., Yosef N., Thalhamer T., Zhu C., Xiao S., Kishi Y., Regev A., Kuchroo V.K. (2013). Induction of pathogenic TH17 cells by inducible salt-sensing kinase SGK1. Nature.

[40] Hagiwara A., Bydder M., Oughourlian T.C., Yao J., Salamon N., Jahan R., Villablanca J.P., Enzmann D.R., Ellingson B.M. (2021). Sodium MR Neuroimaging. AJNR Am. J. Neuroradiol..

[41] Poku L.O., Phil M., Cheng Y., Wang K., Sun X. (2021). ^23^Na-MRI as a Noninvasive Biomarker for Cancer Diagnosis and Prognosis. J. Magn. Reson. Imaging.

[42] Vesely M.D., Schreiber R.D. (2013). Cancer immunoediting: Antigens, mechanisms, and implications to cancer immunotherapy. Ann. N. Y. Acad. Sci..

[43] Song K., Farzaneh M. (2021). Signaling pathways governing breast cancer stem cells behavior. Stem Cell Res. Ther..

[44] Zhang X., Powell K., Li L. (2020). Breast Cancer Stem Cells: Biomarkers, Identification and Isolation Methods, Regulating Mechanisms, Cellular Origin, and Beyond. Cancers.

[45] Wojtukiewicz M.Z., Rek M.M., Karpowicz K., Gorska M., Politynska B., Wojtukiewicz A.M., Moniuszko M., Radziwon P., Tucker S.C., Honn K.V. (2021). Inhibitors of immune checkpoints-PD-1, PD-L1, CTLA-4-new opportunities for cancer patients and a new challenge for internists and general practitioners. Cancer Metastasis Rev..

[46] Babamohamadi M., Mohammadi N., Faryadi E., Haddadi M., Merati A., Ghobadinezhad F., Amirian R., Izadi Z., Hadjati J. (2024). Anti-CTLA-4 nanobody as a promising approach in cancer immunotherapy. Cell Death Dis..

[47] Varricchi G., Galdiero M.R., Marone G., Criscuolo G., Triassi M., Bonaduce D., Marone G., Tocchetti C.G. (2017). Cardiotoxicity of immune checkpoint inhibitors. ESMO Open.

[48] Liberti M.V., Locasale J.W. (2016). The Warburg Effect: How Does it Benefit Cancer Cells?. Trends Biochem. Sci..

[49] Liu G., Luo Q., Li H., Liu Q., Ju Y., Song G. (2020). Increased Oxidative Phosphorylation Is Required for Stemness Maintenance in Liver Cancer Stem Cells from Hepatocellular Carcinoma Cell Line HCCLM3 Cells. Int. J. Mol. Sci..

